# Effects of freezing storage on the DNA extraction and microbial evaluation from anaerobic digested sludges

**DOI:** 10.1186/s13104-015-1407-2

**Published:** 2015-09-07

**Authors:** Valeria Romanazzi, Deborah Traversi, Eugenio Lorenzi, Giorgio Gilli

**Affiliations:** Hygiene Division, Department of Public Health and Pediatrics, University of Torino, Via Santena 5 bis, 10126 Turin, Italy; SMAT, Società Metropolitana Acque Torino S.p.A., Corso XI Febbraio 14, 10152 Turin, Italy

**Keywords:** Sludge, Wastewater treatment, Biotechnology, DNA extraction, PCR (polymerase chain reaction)

## Abstract

**Background:**

The anaerobic digestion is one of the most spread renewable energy technology. The input biomasses included various environmental problematic wastes such as sludge coming from wastewater treatment plant (WWTP) and organic fraction of municipal solid waste (OFMSW). As biomolecular procedures have become important tools for the microbial characterisation of anaerobic samples coming from the reactors, it is crucial sampling and extracting properly DNA in order to employ such types of techniques. The current study is aimed to evaluate how freezing temperature and length of storage at −20 °C influence both the extracted DNA yield and microbial community quantifications from digested sludge samples collected at full-scale plants.

**Results:**

From WWTP sludge samples, we observed a reduction of DNA concentration comparing fresh and stored samples for 10 days at −20 °C (ANOVA test *p* < 0.0001), with an estimated DNA loss of approximately 65 % for such types of samples, however the methanogen communities can be assessed respecting the fresh conditions. From OFMSW sludge samples, we observed a reduction in extracted DNA (−90 %), after 120 frozen days, while microbial communities are determined respecting the fresh conditions within 2 months of frozen storage.

**Conclusions:**

The remarkable effect of frozen storage on sludge samples suggests as the better procedure to perform the DNA extraction from fresh sample. On the other hand it is not generally possible, so approximately 2 months of storage at −20 °C appears to be suitable time at which DNA concentrations remain sufficient to perform coherent microbial characterization through quantitative qRT-PCR.

## Background

Solid organic waste removal has become an ecological problem that is increasingly being recognized as the result of an increase in public health concerns and environmental awareness. The organic fraction of solid waste has been acknowledged as a valuable resource that can be converted into useful products through microbial transformations [1]: in this frame the anaerobic treatment seems to be one of the most promising approaches [2–5]. Understanding microbial population development in landfills over a length of time is challenging due to the complexity of waste materials deposited and the spatial heterogeneity of landfills. Considerable efforts have been directed toward the development of rapid and dependable methods for the detection and enumeration of microbial populations, such as methanogens [6], sulphate-reducing bacteria [7] and hydrolytic microorganisms [8] in different types of complex environmental matrices, such as soil, sediment, sludge and stool [9–11]. The molecular techniques for the characterisation and quantification of single microbial communities—with quantitatively and qualitatively sufficient extracted DNA—are now quite widespread for the optimization of the biogas quantity and quality. The complexity of matrices such as those described above, together with storage time, can interfere with some technical aspects, making these types of samples difficult to treat. Storage conditions are of great importance when they are related to the quantification of microbial communities characterizing the anaerobic sludges especially at full-scale digesters where there isn’t a own biomolecular laboratory. Studies on microbial characterization of digested sludges are recently growing, since it is considered as an important bioindicator of the state of bioreactor functioning in which the anaerobic digestion process is realized [4, 6, 12–14]. Since the effects of storage conditions on microbial community are likely expected, it should be relevant to investigate the freezing effects on microbial population. Various results concerning the effects of handling and storage conditions on DNA and on the microbial characterization from different environmental matrices exist [10, 11, 15–17], but information regarding the storage effects of anaerobic digested sludge samples are still lacking. Various studies revealed that the phylogenetic structure of the microbiota did not significantly differ when human or environmental samples were stored at different temperatures [15–17]. A lower stability of DNA was observed only for storage at room temperatures after 24 h [16].

On the other hand, it is important to recognize that studies on the phylogenetic structure of the microbial populations require qualitative evaluations when referring to the relative composition of the microbiota, whereas quantitative measurements using a standard curve represent a different tool that is able to provide quantitative information [18]. Therefore, the current study aimed to define the influence of technical conditions—including freezing and storage time—on extracted DNA yield and on microbial targets such as total bacteria and methanogens. The study was carried out on full-scale anaerobic digesters of wastes including digested sludge samples coming from a wastewater treatment plant (WWTP) and from the organic fraction of municipal solid waste (OFMSW).

## Results and discussion

### WWTP samples

**Fig. 1:**
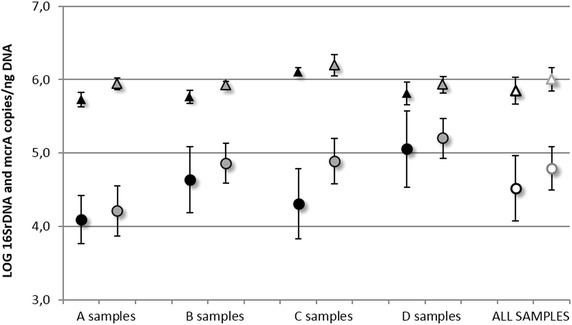
Total bacteria (*triangles*) and methanogens (*dots*) in the four fresh and frozen sample types. *Black* and *grey* colours indicate DNA extraction from fresh sample (first extraction) and from 10 days-frozen storage samples, respectively. (*A samples* input secondary WWTP sludge; *B samples* output secondary WWTP sludge; *C samples* input mixed WWTP sludge; *D samples* output mixed WWTP sludge)

Mean of DNA concentrations in the A, B, C and D samples are reported in Table 1, comparing two DNA extraction time points. Samples stored for 10 days at −20 °C displayed a significant decrease of DNA concentrations respect to fresh samples (ANOVA p < 0.0001) of nearly 63 % (T test p = 0.001), 58 %, (T test p < 0.0001), 72 % (T test p < 0.0001) and 70 % (T test p = 0.001) for the A, B, C and D digested sludge types, respectively. Considering fresh samples and comparing them by influent and effluent, no significant decrease in the DNA concentrations is observed for samples from the secondary treatment (A vs B samples, T test p = 0.125, about 26 % decrement), and no significant increase in the DNA concentrations is observed for samples from mixed treatment (C vs D samples, T test p = 0.120, about 28 % increment). Considering quantifications of microbial communities, total Bacteria have a statistically significant decrement in effluent samples when comparing samples from C and D mixed sludges (−0.05 % in D samples, T test p < 0.0001). However, considering the anaerobic microbial community, a significant increased concentration of methanogens is observed in effluent samples for both of the two treatment systems (A vs B secondary sludge samples T test p = 0.04; C vs D mixed sludge samples T test p < 0.0001) (Fig. 1). Considering frozen samples and comparing them by influent and effluent, a not significant decrement in the DNA concentrations of secondary sludges is observed (A vs B samples, T test p = 0.464, about 16 % decrement), and a not significant increment in the DNA concentrations of mixed sludges is attested (C vs D samples, T test p = 0.069, about 35 % increment). In this case when considering the total bacterial microbial community, a statistically significant decrement in effluent samples was only found in mixed sludges (−0.04 % in D samples, T test p = 0.001 comparing C and D mixed samples), meanwhile concerning the anaerobic microbial community, a significant increased concentration of methanogens is observed in effluent samples for both of the two treatment systems (A vs B secondary sludge samples +13 % in B samples, T test p < 0.0001; C vs D mixed sludge samples +0.06 % in D samples, T test p = 0.007) (Fig. 1). Both for total bacteria and for methanogens concentrations—expressed as extracted DNA mass unit—were similar comparing fresh and frozen quantifications for the same kind of sample, moving into the same order of magnitude (Log scale). The positive and significant correlation between quantifications from the two extraction points reinforces this last evidence (for total bacteria Spearman’s rho = 0.662, p < 0.0001; for methanogens Spearman’s rho = 0.813, p < 0.0001).

**Table 1:** Comparison of DNA concentrations in anaerobic sludges from WWTP and OFMSW samples in the two experimental line

	DNA µg/g of weighted sample ± SD	
WWTP		
	Fresh samples	Frozen samples
A samples	246.1 ± 107.7	89.6 ± 48.2
B samples	182.0 ± 65.2	75.5 ± 35.4
C samples	103.2 ± 44.6	28.3 ± 12.4
D samples	143.9 ± 64.9	43.5 ± 21.1
All samples	168.8 ± 89.0	59.2 ± 39.7

OFMSW		
Days elapsed	E sample	F sample
1	21.6	32.36
19	9.16	22.8
28	13.64	35.88
29	4.64	12.36
21	7.32	15.84
27	4	2.36

The mean values and SD are reported, based on 10 replicates for each type of samples, concerning WWTP samples. For OFMSW experimental line, only two samples were available, so SD was not calculated, since E and F samples were independent sludge samples

**Fig. 2:**
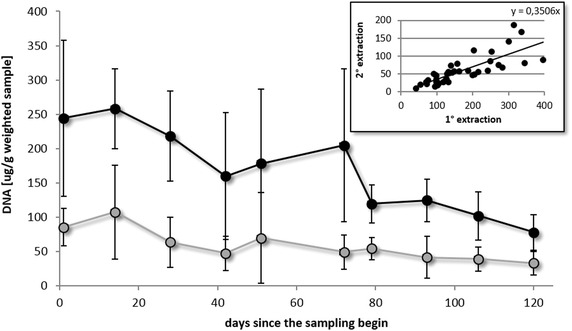
Correlation between the two DNA extraction time point batches. Each data point refers to the mean value of the DNA concentration of the four types of samples collected during one sampling day. In the *small graph* the slope value corresponds to the preserved amount of DNA from both of the extraction time points. In the extended *graph black line* and *dots* represent the DNA yields in fresh samples, *grey line* and *dots* represent the DNA yields in 10-days frozen samples, finally the *dashed line* represents the DNA concentration tendency with the applied correction factor

Using a correlation model between the two time points of DNA extraction, the slope value resulting from the equation (=0.35) provides an indirect indication of the amount of DNA lost due to the frozen storage (nearly 65 %) (Fig. 2). We have applied this value on DNA concentrations from ten days frozen samples, observing successfully that the normal decrease of DNA concentration occurring in frozen samples can be adequately controlled.

### OFMSW samples

Considering the two E and F samples, their initial DNA concentrations reported on the first monitoring day were 21.6 and 32.4 µg/g dry weight sample, respectively. The noticeable reduction of the total amount of DNA during frozen storage, as observed throughout nearly 4 months of repeated extractions, is evident for both of samples (Table 1). The tendency does not display a regular decrease, however the mean value of the quantified DNA concentration is inversely proportional to the length of the frozen storage period (Spearman’s rho = −0.886, p = 0.019). Overall, the total lost DNA quantity is 81 and 93 % for E and F samples, respectively, when comparing the mean of the first and the last time point DNA concentrations (T test, p = 0.049). Moreover, the linear regression between the DNA concentration, as evaluated for the two samples, and the frozen storage time displays a decrease of approximately 6.6 % for every 10 days of storage.

**Fig. 3:**
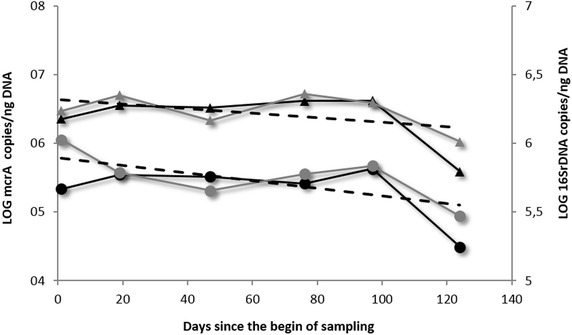
Total bacteria (*triangles*) and methanogens (*dots*) enumeration in the two OFMSW samples, named E sample (*grey line*) anf F sample (*black line*). M The quantification of both the microbial targets was performed at regular intervals (meanly every 20 days) in order to monitor the effect of the storage at −20 °C along the time

Figure 3 shows the presence of total bacteria and methanogens in E and F samples as a quantification of the *16SrDNA* and *mcrA* functional gene target respectively. Both total bacteria and the methanogens are quantifiable throughout the entire average abundance of the monitoring period with an average of approximately 10^6^*16SrDNA* and 10^5^*mcrA* copies number µl^−1^ of DNA extracts, and the gene target concentrations in the two samples are quite similar (paired T test p = 0294, and p = 0.160 for total bacteria and methanogens respectively). Overall, the mean of the methanogen quantification in the two samples is inversely proportional to the length of frozen storage (Spearman’s rho = −0.600, p = 0.208), for total bacteria the levels seem to be less affected by storage time. In particular, the value of the microbial quantifications display a constant tendency until extracted DNA concentrations are appreciable. Thus, after approximately 100 days of frozen storage, in addition to the reduction of DNA concentration, there was a nearly two orders of magnitude reduction in the *mcrA* quantification value, while only about an half a log for total bacteria.

Since the advent of DNA-based methods to characterise complex bacterial communities in biological and/or environmental samples, often freezing samples is required, in order to perform the analysis on a larger number of samples collected at different times. This is crucial especially switching from lab scale to full scale digesters. The use of specific biomolecular techniques has been recently expanding in the field of microbial characterisation of environmental samples in addition to the use of the classical and time-consuming culture methods. Even if these techniques are still very reliable with a low DNA concentration, it is important to take into account that also the technical aspects such as storage conditions could have a role in obtaining higher quality-genomic samples.

Concerning our study, variations of DNA concentrations among the four types of WWTP fresh samples are expected, since different microbial communities are temporally selected during the anaerobic digestion process. In particular the anaerobic conditions establishing within the bioreactor induce the growth of the anaerobic microbial communities, leading to increased DNA concentrations and to increased presence of growing methanogens in the effluent samples (B and D samples). While this tendency is well evident for the mixed treatment, the secondary system shows an inverse tendency likely due to inhibition of aerobic community (typically more abundant in secondary activated sludges than mixed sludges) during the anaerobic digestion, thus masking the contribution of growing anaerobic communities (Table 1). In fact when considering only the methanogens, an increased concentration of anaerobic communities is observed in effluent samples for both of the two treatment systems (Fig. 1). Even if—comparing fresh and frozen samples—all the fluctuations in terms of DNA concentrations are kept, the significant reduction found in the frozen samples pays serious attention to the microbial quantification. As observed for OFMSW samples, the methanogens quantification remains quite constant for DNA concentrations ranging from 4.6 to 35.9 µg g^−1^ of weighted sample: only for DNA concentration values below this range, also the methanogens quantification has a decrement but not statistically significant, probably attributable to the low numerosity of data (Fig. 3) suggesting the DNA extraction from such samples after short freezing time. A 2 months period is desirable. Storage conditions are considered to be a critical component of DNA-based microbial community analysis methods. In regards to our study, since fresh and frozen samples were technically processed in a same way (same DNA extraction kit, same operative DNA extraction conditions), the observed differences in the DNA concentrations comparing fresh and frozen samples could be mainly likely due to the freezing storage, considering also the intrinsic variability of a complex matrix such as anaerobic sludge. Results highlight neither the freezing temperatures nor the length of frozen storage (ranging from 10 days to approximately 2 months) appeared to adequately preserve environmental samples from a genomic DNA yield point of view. It should be also important to distinguish between “freezing step” and “thawing step” among the general expression “freezing storage”. Actually we are not really able to attribute the observed differences in DNA yields to the first or to the second step. However the Table 1—concerning DNA quantity in OFMSW samples—shows how if the problem was the thawing, a decrease in DNA yield over the time should not be observed, and the last aliquot should have a DNA content comparable to the first aliquot, since the DNA degradation (by DNAse or other) should occur with the same entity on the first aliquot as well as on the last one. For this reason the degradation seems to be likely attributable to the length of time during which the sample is kept at −20 °C, and not to the thawing phase. Freezing storage is only one among the many troubles related to the treatment of such complex type of samples. Actually literature on anaerobic sludge samples do not contemplate, for example, the use of any substances to preserve DNA during the thawing. During this phase microbial cells could be broken and DNA as well as DNAases that destroy DNA could be released. However, similar studies on this kind of sample indicate any substance or reagent to preserve the anaerobic sludge samples from the degradation of nucleic acids [4, 6, 12, 14, 19]. One of the major impediments in quantifying microorganisms from environmental samples is, for example, the presence of several potentially PCR-inhibiting factors, including humic substances, metals and ions that in the present study were adequately controlled by ten-fold diluting of samples [4, 6, 20]. Also there are other several wastewater treatment process parameters can influence the presence and the viability of microbial communities in these samples: retention time, type of digestion (batch or continuous), reactor configuration, pH, concentration and composition of (volatile) fatty acids, available nutrients, structure and composition of bacterial communities, microbial competition and chemical interactions, including ammonia toxicity [19, 21, 22], could interfere both with the DNA quantification and the molecular characterisation of microbial communities, making this type of sample much more variable from a management point of view than other environmental and biological samples originated in more stable conditions. In line with freezing storage timing tested on OFMSW samples, the methanogens quantification in WWTP samples is not so affected when performed on ten-days frozen samples, comparing it with the quantification on fresh samples (Fig. 1).

We quantified the methanogen community as an example of a typical microbial community occurring in this type of sample, in which the anaerobic digestion process is intended for biogas production. Theoretically, no differences are expected concerning the structure of the microbial community, as the freezing storage—DNA reduction occurs proportionally for the total DNA. Temperature plays a primary role in the stability and selection of both the identity of individual species and the overall bacterial diversity supported by a wastewater treatment reactor [23–25]. Freezing storage of this kind of sample could be a further variable in the evaluation of microbial communities.

## Conclusions

Our findings indicate that the enumeration of abundant microbial populations from complex samples—such as methanogens in anaerobic sludges—was not significantly affected when processed after short periods of storage at −20 °C (about 10 days), comparing with fresh-processed samples. Also longer time storage at −20 °C does not significantly affect the DNA concentration nor the principal microbial communities colonizing anaerobic sludge samples. Although the freezing storage represents a consolidated lab practice to preserve samples, our purpose was to consider how this technical procedure potentially could lead to underestimated results; this is relevant when the quantification and characterisation of the microorganism community in reactors—together with chemical and physical parameters—is used for the control and management of the anaerobic process.

## Methods

### Samples

Two different types of sampling activities and schedule procedures were performed (Table 2). For the first experimental line, sludge samples coming from the anaerobic digestion treatment of WWTP sludge were intended to highlight any differences in DNA yield between fresh and 10-days frozen samples. Forty samples were provided by the SMAT (Società Metropolitana Acque Torino S.p.A.), which is concerned with wastewater treatment in Castiglione Torinese (Italy). Samples coming from two different biodigesters: one biodigester is fed with sludge from the secondary treatment of wastewater (A and B samples), and another is fed with mixed sludge (50 % of sludge from primary treatment + 50 % of sludge from secondary treatment; C and D samples), where A and C samples were collected at the influent (feeding input sludge), and the B and D samples were collected at the effluent (digested output sludge) of each biodigester. For each sample type (A, B, C and D), 10 samples were collected, every 15 days along a period of 5 months. 500 ml of sludge sample were collected in sterile PET bottles for microbiological analysis, and then the samples were divided into aliquots of 50 ml of undiluted sludge. Thus, for each sample, DNA was extracted twice: the first extraction was intended to quantify DNA from fresh sample, and the second extraction occurred after 10 days of frozen storage at −20 °C using the same aliquot.

**Table 2:** Schematic experimental design of the two conducted experiments in which two clearly distinct activities are described

Aim	Samples characteristics	Sample origin	Schedule of DNA extraction
Comparing the extracted DNA concentration in fresh and frozen samples	4 types of samples named A, B, C and D	Sludge from anaerobic digestion of WWTP	For each sample: DNA extraction from fresh sample and DNA extraction from 10-days frozen sample
Verifying the preservation of the extracted DNA concentration in frozen samples	2 samples, named E and F	Sludge from anaerobic digestion of OFMSW	Repeated DNA extractions from aliquots of the same sample after different storage periods

For the second experimental line, sludge samples coming from the anaerobic digestion of OFMSW treatment plant in the province of Alessandria (Italy) were used to test the DNA preservation status of saved samples throughout a frozen storage period ranging from 20 to 120 days. Sludge samples were harvested from two different sections of the same sole biodigester under investigation: considering a reactor diameter of approximately 25 m, the E sample was harvested from a more external side (2 m section from the wall towards the centre), and the F sample was harvested from the central section of the reactor. The sludge samples were collected in the same manner of the WWTP samples; after an accurate mixing, undiluted samples were divided into 10 aliquots and then stored at −20 °C. Arranging a number of separate aliquots for each sludge sample was also useful to correctly perform the following biological evaluations: in this way thawing and DNA extraction occurred one sole time for each aliquot, avoiding repeated thawing and re-freezing steps. With a regular periodicity (on average every 25 days, for a entire period of approximately 4 months), each of these stored aliquots was thawed for DNA extraction and quantification until the DNA concentration reached the threshold of 1 µg ml^−1^. This threshold is the minimum DNA concentration needed to provide sufficient DNA template for each qPCR reaction [26] and to detect single microbiological groups, such as methanogens, for which at least 0.1 ng of DNA for PCR is necessary [13].

### Extraction, quantity and quality of DNA

50 ml of sludge sample from each type of biodigester were centrifuged at 2500*g* for 10 min, to collect all particulate matter eventually in the supernatant (including cells and bacteria) into the pellet at the bottom, and the supernatant was then discarded. Every DNA extraction was performed with a commercial kit already employed for sludge samples [7, 15] (PowerSoil DNA Isolation Kit, MO-BIO Laboratories Inc., Carlsbad), starting from 0.25 g of semi-dry pellet, in accordance to the manufacturer’s instructions. No treatment to protect DNA against DNAse was applied neither for fresh nor for frozen-thawed samples: the same DNA extraction procedural conditions for all samples coming from both of the two experimental lines were carried out. The fluorimetric quantification of each DNA sample was performed using a Qubit™ Fluorometer and Qubit™ dsDNA HS Assay by Invitrogen (distributed by Life Technology Ltd., Paisley, UK) on diluted DNA aliquots (range of standard curve: 0–10 µg ml^−1^). The DNA samples were stored at −20 °C until the PCR analysis was performed. The A260/280 ratio was calculated by spectrophotometric method (BioTek Instruments plate Reader—PowerWave HT, accessorized with Gen5 software) for the quality of extracted DNA. For the WWTP freshly-processed samples the average ratio was 1.95, conversely the average ratio is less favorable for frozen WWTP samples with a value of 1.8.

### RT-qPCR for microbial target quantification

Real-Time Quantitative PCR (RT-qPCR) was performed using a Chromo4 thermal-cycler (Bio-Rad, Hercules, CA) and Opticon Monitor 3 Software. The amplification target for total bacteria quantification was the ribosomal RNA 16S subunit (*16SrDNA*) [27]; for methanogen quantification was the functional gene methyl-coenzyme M reductase α-subunit (*mcrA*) [6]. For methanogens 2 µl of ten-fold diluted sample (about 8 ng of DNA per single PCR reaction) was added to the reaction mixture consisting of 10 µl of SsoFast EvaGreen^®^ Supermix (Bio-Rad, Hercules, CA), 0.25 µmol l^−1^ of forward (*mlas*-F: 5′-GGTGGTGTMGGDTTCACMCARTA-3′) and reverse (*mcrA*-R: 5′-CGTTCATBGCGTAGTTVGGRTAGT-3′) primers [6] (Thermo Fisher Scientific, Waltham, MA, USA), and 7 µl of ultrapure water in a 20 µl volume reaction. Primers targeting the specific portion of DNA coding for the functional gene *mcrA* were used as previously described by Steinberg and Regan [6] (the accession numbers of the used sequences are reported by Steinberg and Regan in their study). Total methanogens were quantified using a standard curve in which the *mcrA* gene from *Methanosarcina acetivorans* was placed into the pCR21 vector [6]. The *mcrA* plasmid was first amplified through the transformation of Top 10 *E. Coli* cells, and then, the plasmid was extracted using a commercial kit (NucleoSpin Plasmid, Macherey–Nagel, Düren, Germany) as previously described [13]. Serial ten-fold dilutions of the plasmid DNA containing *mcrA* gene was applied, and a six-point standard curve ranging from 2.5 × 10^7^ to 2.5 × 10^2^ copies of *mcrA* was applied. For total bacteria 2 µl of ten-fold diluted samples was added to reaction mixture consisting of 8 µl IQ™ Multiplex PowerMix (Bio-Rad, Hercules, CA, USA), 0.2 µl molecular probe (10 µM concentration), 0.5 µl each of the forward and reverse primers (10 µM final concentration, Thermo Fisher Scientific, Waltham, MA, USA) and 8.8 µl of ultrapure water in a 20 µl final reaction volume. To obtain an absolute quantification of all targets in sludge samples, the genomic DNA of each microorganism, provided by the American Type Culture Collection, LGC (ATCC, Manassas, VA, USA), was used as standards. Serial ten-fold dilutions of each ATCC standard were assayed, and quantifications are expressed as gene copy number µl^−1^ of extracted DNA, assuming four *16SrDNA* gene copies per bacterium [4]. Standards and samples were tested in triplicate and DNA quality and integrity were evaluated by gel electrophoresis before the chain reaction. Reaction condition were 95 °C for 3 min (1×), then 95 °C for 3 s, 55 °C for 45 s, 72 °C for 30 s and 83 °C for 5 s (40×). A final melt curve analysis was performed to verifying the specificity of PCR products. The program was as follows: denaturation of 1 min at 95 °C, cooling of 1 min at 65 °C and then 95 °C, at a rate of 0.5 °C per cycle. For total bacteria, the reaction conditions were 95 °C for 3 min (1×), then 95 °C for 30 s, 55 °C for 1 min (39×). The other amplifications were performed for 30 s, 55 °C for 1 min (40×); a melt curve was not performed. The results were expressed as the *mcrA* and *16SrDNA* gene copies number *per* ng of DNA. The triplicate averages were accepted only if the coefficient of variation was below 20 %. All the PCRs were conducted taking into account the reaction efficiency (ŋ efficiency values >75 %).

### Statistical analysis

The Spearman correlation coefficient was used to assess the relationship between the variables. Considered variables were the DNA yield expressed as ug g^−1^ of weighted sample, and microbial targets expressed as gene copies number per ng of DNA. A T test of independent variables and an ANOVA test were used to compare two or more means, respectively. A paired T-test was used to compare singular values from two sets of data. Log-transformation was needed for the microbial evaluation data. All the statistical evaluations were considered significant at p < 0.05. Statistical analyses were performed with the SPSS Package version 19.0 for Windows.

## Abbreviations

WWTP: wastewater treatment plant
OFMSW: organic fraction municipal solid waste
RT-qPCR: real time quantitative polymerase chain reaction
*mcrA*: methyl-coenzyme M reductase α-subunit gene

## References

[1] Khalid A, Arshad M, Anjum M, Mahmood T, Dawson L (2011). The anaerobic digestion of solid organic waste. Waste Manag.

[2] Diaz I, Donoso-Bravo A, Fdz-Polanco M (2011). Effect of microaerobic conditions on the degradation kinetics of cellulose. Bioresour Technol.

[3] Guerrero L, Chamy R, Jeison D, Montalvo S, Huilinir C (2013). Behavior of the anaerobic treatment of tannery wastewater at different initial pH values and sulfate concentrations. J Environ Sci Health Tox Hazard Subst Environ Eng.

[4] Merlino G, Rizzi A, Villa F, Sorlini C, Brambilla M, Navarotto P, Bertazzoni B, Zagni M, Araldi F, Daffonchio D (2012). Shifts of microbial community structure during anaerobic digestion of agro-industrial energetic crops and food industry byproducts. J Chem Technol Biotechnol.

[5] Ryckebosch E, Drouillon M, Veruaeren H (2011). Techniques for transformation of biogas to biomethane. Biomass Bioenerg.

[6] Steinberg LM, Regan JM (2008). Phylogenetic comparison of the methanogenic communities from an acidic, oligotrophic fen and an anaerobic digester treating municipal wastewater sludge. Appl Environ Microbiol.

[7] Ben-Dov E, Brenner A, Kushmaro A (2007). Quantification of sulfate-reducing bacteria in industrial wastewater, by real-time polymerase chain reaction (PCR) using dsrA and apsA genes. Microb Ecol.

[8] Regueiro L, Veiga P, Figueroa M, Alonso-Gutierrez J, Stams AJ, Lema JM, Carballa M (2012). Relationship between microbial activity and microbial community structure in six full-scale anaerobic digesters. Microbiol Res.

[9] Pesaro M, Widmer F (2006). Identification and specific detection of a novel pseudomonadaceae cluster associated with soils from winter wheat plots of a long-term agricultural field experiment. Appl Env Microbiol.

[10] Rissanen AJ, Kurhela E, Aho T, Oittinen T, Tiirola M (2010). Storage of environmental samples for guaranteeing nucleic acid yields for molecular microbiological studies. Appl Microbiol Biotechnol.

[11] Wallenius K, Rita H, Simpanen S, Mikkonen A, Niemi RM (2010). Sample storage for soil enzyme activity and bacterial community profiles. J Microbiol Methods.

[12] Garcia-Pena EI, Parameswaran P, Kang DW, Canul-Chan M, Krajmalnik-Brown R (2011). Anaerobic digestion and co-digestion processes of vegetable and fruit residues: process and microbial ecology. Bioresour Technol.

[13] Traversi D, Villa S, Acri M, Pietrangeli B, Degan R, Gilli G (2012). The role of different methanogen groups evaluated by Real-Time qPCR as high-efficiency bioindicators of wet anaerobic co-digestion of organic waste. AMB Express.

[14] Zhao YG, Wang AJ, Ren NQ (2009). Effect of sulfate absence and nitrate addition on bacterial community in a sulfidogenic bioreactor. J Hazard Mater.

[15] Bahl MI, Bergstrom A, Licht TR (2012). Freezing fecal samples prior to DNA extraction affects the Firmicutes to Bacteroidetes ratio determined by downstream quantitative PCR analysis. FEMS Microbiol Lett.

[16] Carroll IM, Ringel-Kulka T, Siddle JP, Klaenhammer TR, Ringel Y (2012). Characterization of the fecal microbiota using high-throughput sequencing reveals a stable microbial community during storage. PLoS One.

[17] Lauber CL, Zhou N, Gordon JI, Knight R, Fierer N (2010). Effect of storage conditions on the assessment of bacterial community structure in soil and human-associated samples. FEMS Microbiol Lett.

[18] Gadkar D, Filion M, Filion M (2012). Studying microbial gene expression in complex environmental matrices using quantitative Real-Time Polymerase Chain Reaction. Quant Real-Time PCR Appl Microbiol.

[19] Farrah SR, Bitton G (1983). Bacterial survival and association with sludge flocs during aerobic and anaerobic digestion of wastewater sludge under laboratory conditions. Appl Env Microbiol.

[20] Merlino G, Rizzi A, Schievano A, Tenca A, Scaglia B, Oberti R, Adani F, Daffonchio D (2013). Microbial community structure and dynamics in two-stage vs single-stage thermophilic anaerobic digestion of mixed swine slurry and market bio-waste. Water Res.

[21] Kearney TE, Larkin MJ, Levett PN (1993). The effect of slurry storage and anaerobic digestion on survival of pathogenic bacteria. J Appl Bacteriol.

[22] Smith SR, Lang NL, Cheung KH, Spanoudaki K (2005). Factors controlling pathogen destruction during anaerobic digestion of biowastes. Waste Manag.

[23] LaPara TM, Nakatsu CH, Pantea LM, Alleman JE (2001). Aerobic biological treatment of a pharmaceutical wastewater: effect of temperature on cod removal and bacterial community development. Water Res.

[24] Konopka A, Zakharova T, LaPara TM (1999). Bacterial function and community structure in reactors treating biopolymers and surfactants at mesophilic and thermophilic temperatures. J Ind Microbiol Biotechnol.

[25] Baghchehsaraee B, Nakhla G, Karamanev D, Margaritis A, Reid G (2008). The effect of heat pretreatment temperature on fermentative hydrogen production using mixed cultures. Int J Hydrog Energy.

[26] Nolvak H, Truu M, Truu J (2012). Evaluation of quantitative real-time PCR workflow modifications on 16S rRNA and tetA gene quantification in environmental samples. Sci Total Env.

[27] Dridi B, Henry M, El Khechine A, Raoult D, Drancourt M (2009). High prevalence of *Methanobrevibacter smithii* and *Methanosphaera stadtmanae* detected in the human gut using an improved DNA detection protocol. Plos One.

